# Supplementary material for: Segmentation and characterization of macerated fibers and vessels using deep learning
Source: Plant Methods. 2024 Aug 14;20:126. doi: 10.1186/s13007-024-01244-w (PMC11325806; doi:10.1186/s13007-024-01244-w)
Supplement: Supplementary file 1 — Additional file 1. [file 13007_2024_1244_MOESM1_ESM.pdf]

Deep Learning Approach for Segmentation and Characterization of Fibers and Vessels

Saqib Qamar[1,2,3], Abu Imran Baba[4], Stéphane Verger[2,4,5*], Magnus Andersson[1,2,6*]

[1]Department of Physics, Umeå University, Umeå, Sweden
[2]Integrated Science Lab, Department of Physics, Umeå University, Sweden
[3]Robotics, Perception, and Learning (RPL), Department of Intelligent System, KTH Royal Institute of Technology and Science, Stockholm, 100 44, Sweden.
[4]Umeå Plant Science Centre, Department of Forest Genetics and Plant Physiology, Swedish University of Agricultural Sciences, Umeå, Sweden
[5]Umeå Plant Science Centre (UPSC), Department of Plant Physiology, Umeå University, Umeå, Sweden
[6]Umeå Centre for Microbial Research (UCMR), Umeå, Sweden

Contents

A quick guide to using the model on a Local computer 2
Prerequisites: 2
Installation: 2
Detailed guide to use the model (for beginners and retraining) 3
Installation: 3
Usage: 4
Example: 5
Supporting figures 5
How to use the model in a web application 10

A quick guide to using the model on a Local computer

Prerequisites:

- Conda (https://conda.io/)

- Code repository: https://github.com/sqbqamar/fiberseg

Installation:

1. Download and extract the fiberseg repo to a folder of choice.

2. Open a terminal at or navigate to the repo folder ("…/fiberseg-main/").

3. Create and activate the conda environment with:

```
conda env create -f Fiberseg_env.yml
```

```
conda activate fiberseg
```

<u>Usage:</u>

- Flask (Web app):

  1. Run:

     ```
     python app.py
     ```

  2. Open a web browzer and paste http://0.0.0.0:5000/ in the address bar.
  3. Follow the instructions on screen.

  Note that the results can be downloaded from the links present on the page

- Command line:

  1. As a demo, run:

     ```
     python prediction.py
     ```

This will run the prediction, segmentation and quantification with an example image from the repo and default parameters. See outputs in ("…/fiberseg-main/result/pred_131").

  2. Run the "prediction.py" python script with specific arguments (*See the "detailed guide" for arguments description).* Exemple:

```
python prediction.py --weights best.pt --source 'image/126.jpg' --mask --display --conversion 0.65
```

  See output in ("…/fiberseg-main/result/pred_126").

Example output:

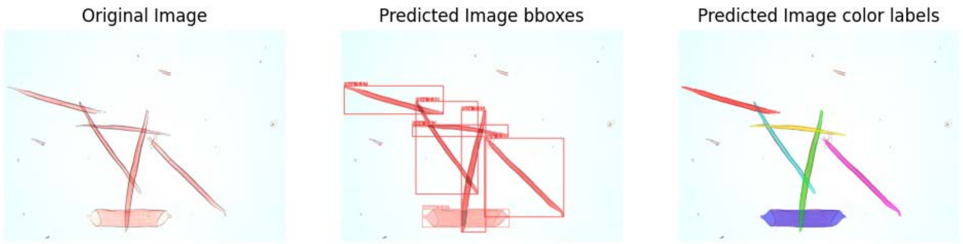

Class Name	Length	Width	Area
Fibre	399,1	24,6935	12935,975
Fibre	412,1	20,0135	11870,3
Vessel	367,9	78	37757,85
Fibre	431,6	24,7	13435,5
Fibre	525,2	28,6	17416,425
Fibre	410,15	22,8735	8629,4

Detailed guide to use the model (for beginners and retraining)

Installation:

1. Install miniconda : Follow the instructions provided here

https://conda.io/projects/conda/en/stable/user-guide/install/index.html#regular-installation

2. Check miniconda installation:

- On Windows, look for the "Miniconda prompt" in the search bar (next to the Windows icon, on the bottom left of the desktop screen).

- On Linux and Mac, open a terminal and copy and paste the commands below into the terminal and press enter.

```
Conda
```

3. Download and extract the "fiberseg" repository to the location of your choice. To do this, click on the green "code" icon on the top right side of the repository page (https://github.com/sqbqamar/fiberseg). Then "Download ZIP". Finally extract the content of the zip to the location of your choice.

4. “Navigate” to the downloaded fiberseg folder (“…/fiberseg-main/”).

- On Linux or Mac you can simply navigate with your regular graphical interface file manager (e.g. Finder or Nautilus) to your “fiberseg-main” folder. Then, right-click on the folder and select “ New terminal at folder” (Mac) or "Open in terminal" (Linux).

- On Windows, open the miniconda prompt (see point 2.) and then navigate to the folder with the "cd" (Change directory) command (https://en.wikipedia.org/wiki/Cd_(command)). For instance, if your fiberseg folder is at C:\Image_Analysis\fiber\fiberseg-main, you should type in the miniconda prompt:

```
cd Image_Analysis\fiber\fiberseg-main
```

Note: If you need to navigate to a different drive (e.g. D:\ instead of C:), first write the name of the drive before the cd commande (i.e. "D: cd \...).

5. Create a new conda environment. To do this, you can directly copy and paste the commands below into the terminal or Miniconda Prompt and press enter.

```
conda env create -f Fiberseg_env.yml
```

Note:This should take a few seconds. This will install python and all the libraries required.

6. Activate the environment. Copy and paste the commands below as in point 5.

```
conda activate fiberseg
```

Now everything should be installed and ready to run.

Usage:

For basic usage, see the quick guide above. The command-line version allows more advanced adjustments, such as using a different trained model, generating and saving the individual binary mask of the segmented cell, using a different conversion factor for the image resolution (pixel size in microns), and displaying or not displaying the segmentation results, in addition to only being saved.

The default command is "`python prediction.py`" but as stated above this will run the prediction on a demo image and with default parameters. Thus our python script takes "arguments" as input to specify which model is used for the segmentation (--*weight*) and on which image it is applied (--*source*). Those are the only two arguments required to run the prediction on your image of choice. Note, however, that the measurement values (length, width, area) is by default calculated in pixel number as well as converted to microns with a default value of 0.65x0.65 microns per pixel. Thus, if working with images that have a different pixel size, you need to specify it to get accurate micron values (--*conversion*).

By default, output data is saved after the segmentation and quantification. All data is saved in the "fiberseg-main/Result" folder, under the folder named "Pred_[ImageName]", where [ImageName] is the name of the original image used for segmentation. Saved data include:

- YOLO prediction image: Full segmentation of the image with overlaid masks and bounding box. **[ImageName]_bboxPred.jpg.**

- Filtered color label image: An image representing the segmentation output, where each label is highlighted in a different color to improve visibility, and labels touching the border (not fully segmentable) have been removed. The remaining labels in this image correspond to the labels for which quantifications are actually performed.

[ImageName]_colorLabels.jpg.

- Quantification results: CSV files containing each object (see [ImageName]_colorLabels.jpg), the cell type, length, width, and area measurement in pixels and in microns.

[ImageName]_summary_micron.csv

[ImageName]_summary_px.csv

In addition to this default output, it is possible to save each segmented/filtered mask individually by adding the --*mask* argument in the command line. Individual images will be saved under

fiberseg-main/Result/Pred_[ImageName]/cmasks/

Finally, by default, the script directly runs the segmentation and measurement and saves the output in the background, but it is also possible to get a pop-up visual output of the original image, YOLO prediction image, and filtered color label image side by side by adding the --display argument in the command line.

List of `python prediction.py` arguments:

--*weight: Path to the trained model.*

--*source: Path to the input image.*

--*mask: Save segmentation masks*

--*display: Display the segmentation results*

--*conversion: Conversion factor for pixel to standard units*

Example:

```
python prediction.py --weights best.pt --source 'image/126.jpg' --mask
--display --conversion 0.65
```

When running this command line from the terminal at the "…/fiberseg-main/" folder, the prediction.py script will use the "best.pt" trained model we provide to segment the image "126.jpg" that is present in "…/fiberseg-main/image" folder, and run the segmentation and quantification. It will additionally save the individual masks, display the output in a pop-up window, and use the conversion factor of 0.65 microns per pixel for quantification output.

Supporting figures

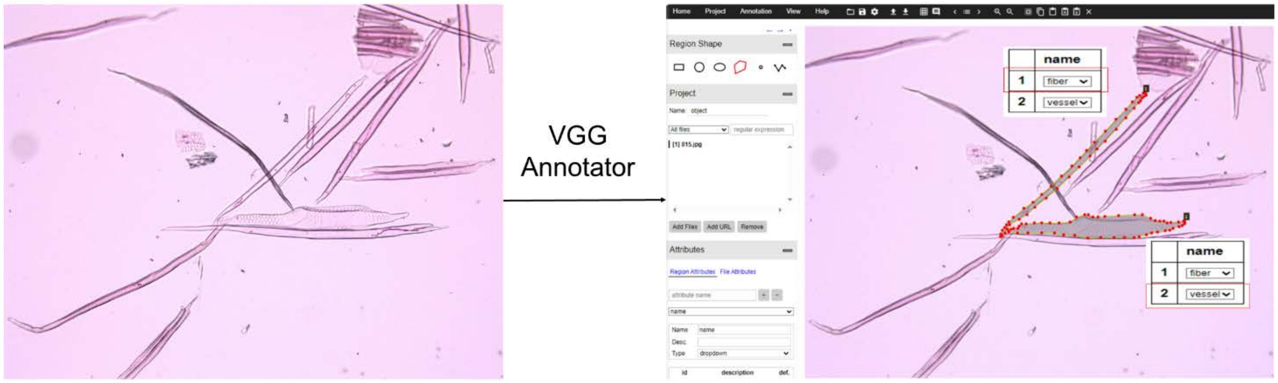

Figure S1. The VGG annotator software was utilized for image annotation, where polygon points are drawn over the area of the object to delineate its shape.

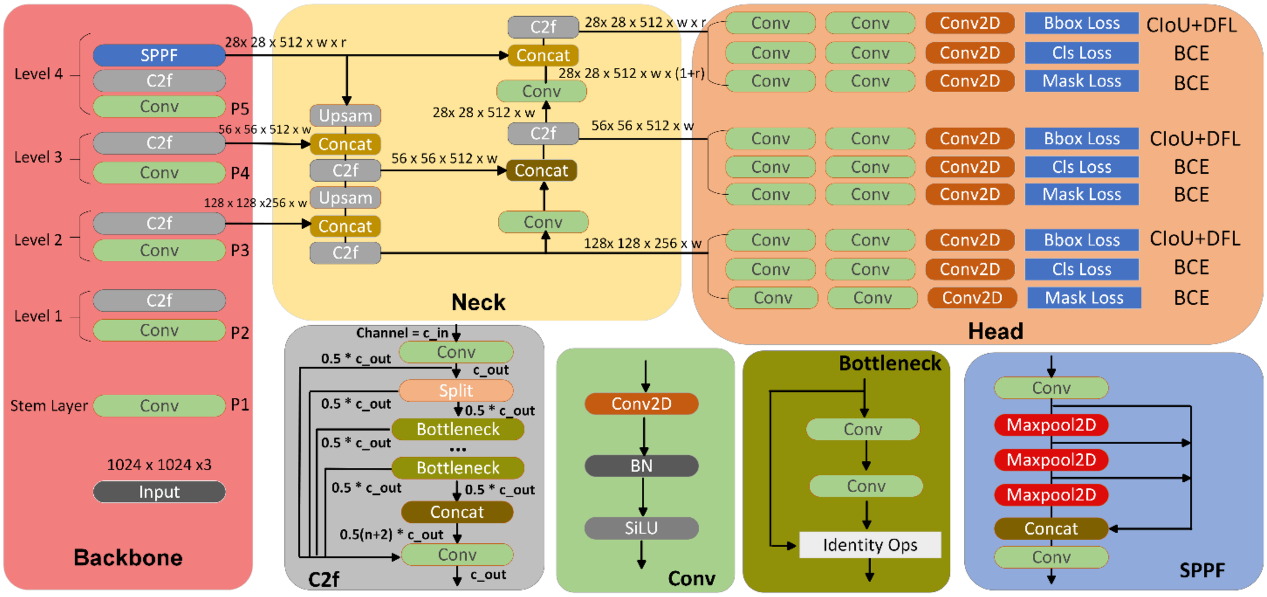

Figure S2. YOLOv8 algorithm consists of four main components: Backbone, Neck, Head, and Loss. Backbone incorporates the Cross Stage Partial (CSP) concept that divides the feature map into two parts: one part applies convolutions, while the other part combines its output with the convolutions from the previous part to enhance performance. Unlike YOLOv5, which uses the C3 module, YOLOv8 replaces it with the C2f module. C2f module comprises two Convolutions and multiple BottleNecks connected through Split and Concat operations. Each Convolution in the C2f module consists of Conv-BN-SiLU. This allows YOLOv8 to capture more complete gradient flow information while keeping the model lightweight. Additionally, YOLOv8 reduces computation by decreasing the number of blocks at each stage compared to YOLOv5. In Level 4, YOLOv8 uses the SPPF module as shown in Figure. SPPF is an improved version of Spatial Pyramid Pooling, designed to increase model inference speed.

To address the problem of loss of important information, it is necessary to incorporate a multiscale fusion of features using architectures like FPN (Feature Pyramid Network) and PAN (Path Aggregation Network). The Neck architecture in the Figure fuses features from different scales. Upper features have more details from extra layers. Lower features keep better location details since they have fewer convolutions. YOLOv8 keeps the FPN and PAN structure. However, it removes convolutions when up sampling to simplify the model.

YOLOv8 splits classification, detection, and masking into separate branches. YOLOv8 introduces an Anchor-Free approach. This method locates the object based on its center and predicts the distance from the center to the bounding box. The YOLOv8-seg algorithm incorporates classification, regression, and mask branches. To train classification and mask branches, it utilizes the cross-entropy loss to minimize the error and improve its prediction performance in our segmentation tasks. The regression branch incorporates two loss functions, namely Distribute Focal Loss (DFL) and CIoU Loss. The Head architecture encapsulates these approaches in the Figure.

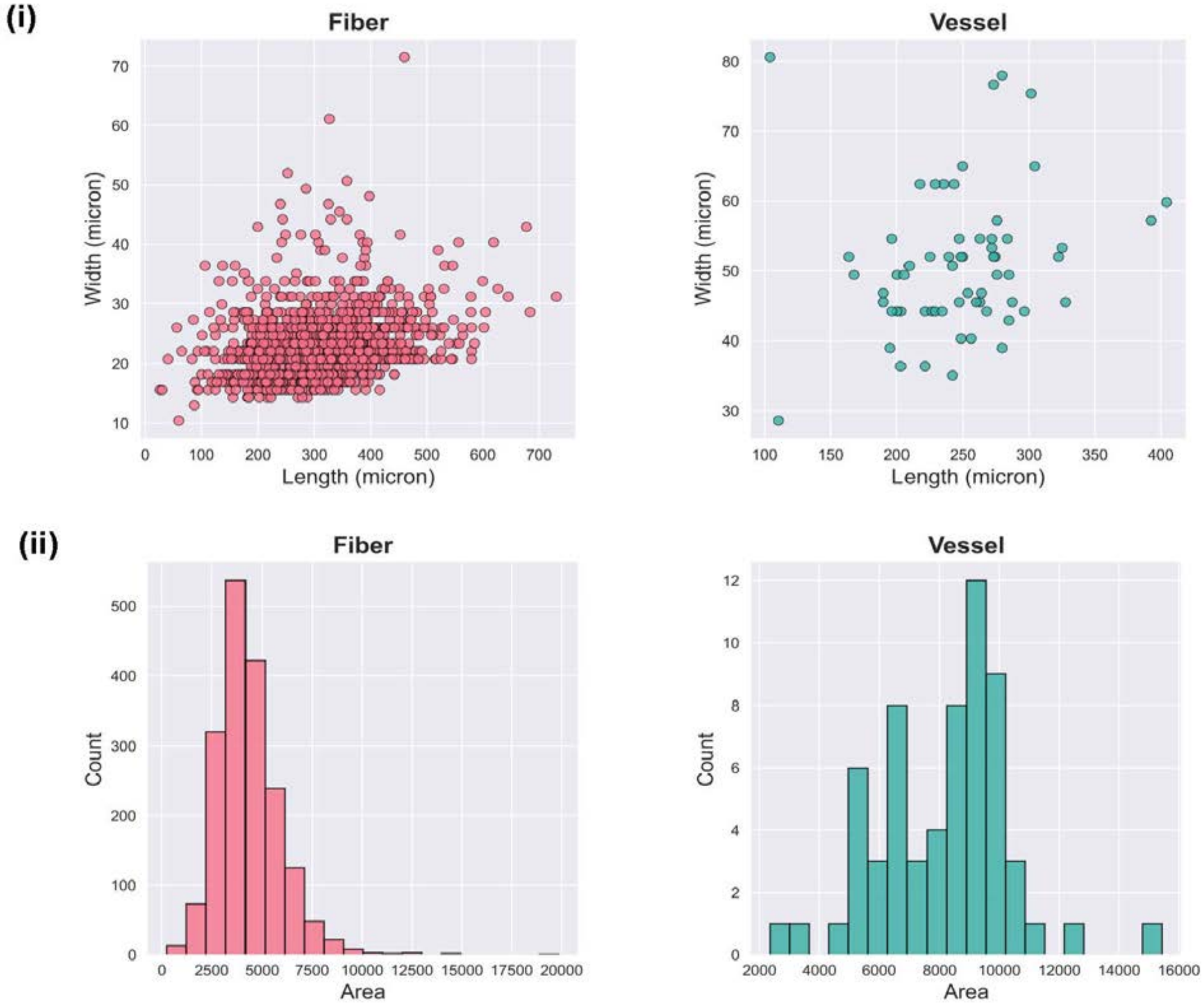

Figure S3. Visualizing distributions and relationships of fibers and vessels in 2 large high-resolution (33,384 x 25,1120) images through **(i)** Scatter Plots of length vs width and **(ii)** Histograms of area distributions. In total, 1818 fibers and 62 vessels are detected.

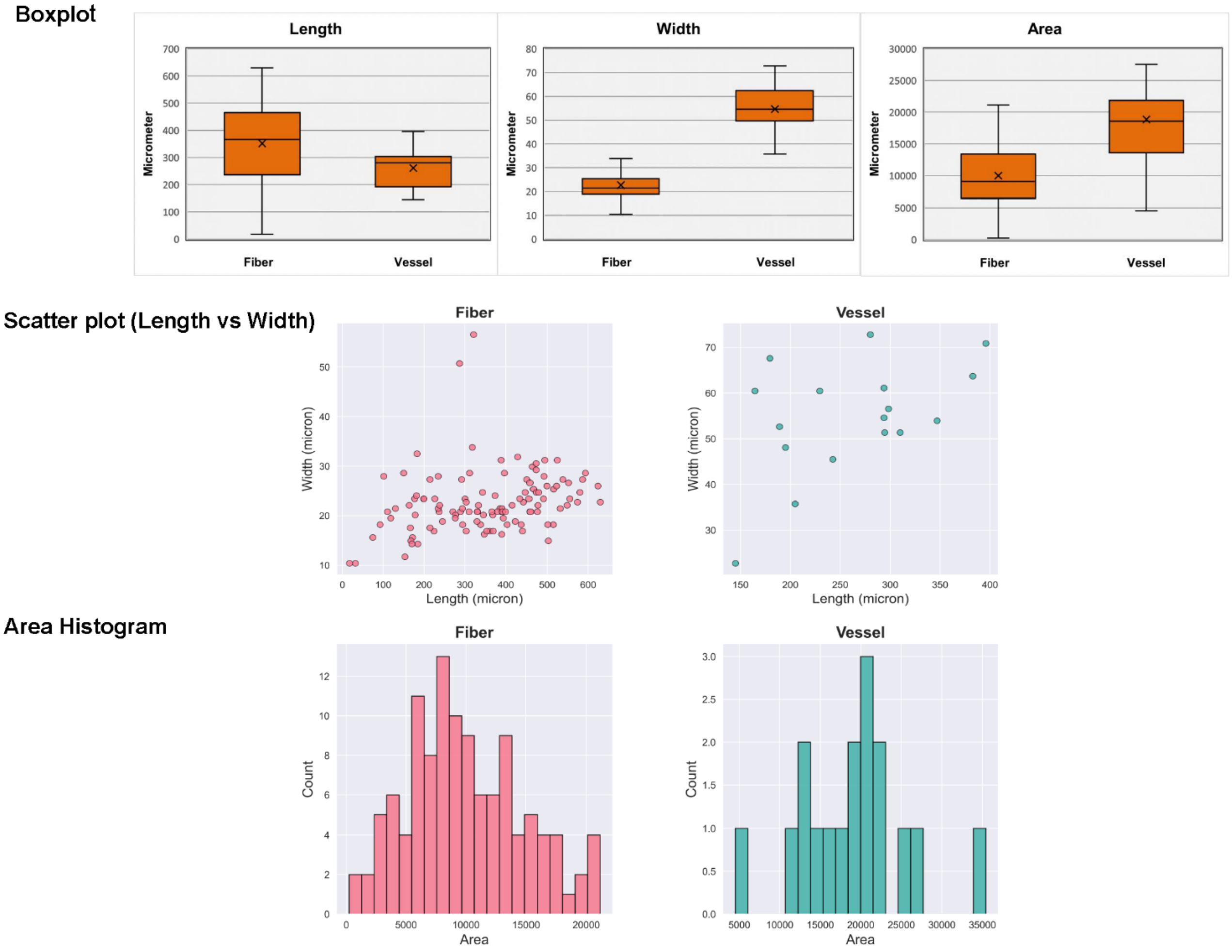

Figure S4. Analyzing fiber and vessel distributions in 20 small images (1920 x 1440). The model maintains performance across different image resolutions. This scale invariance is a crucial property for real-world applications, where varying image dimensions will inevitably be encountered. The total detected fibers and vessels are 115 and 17.

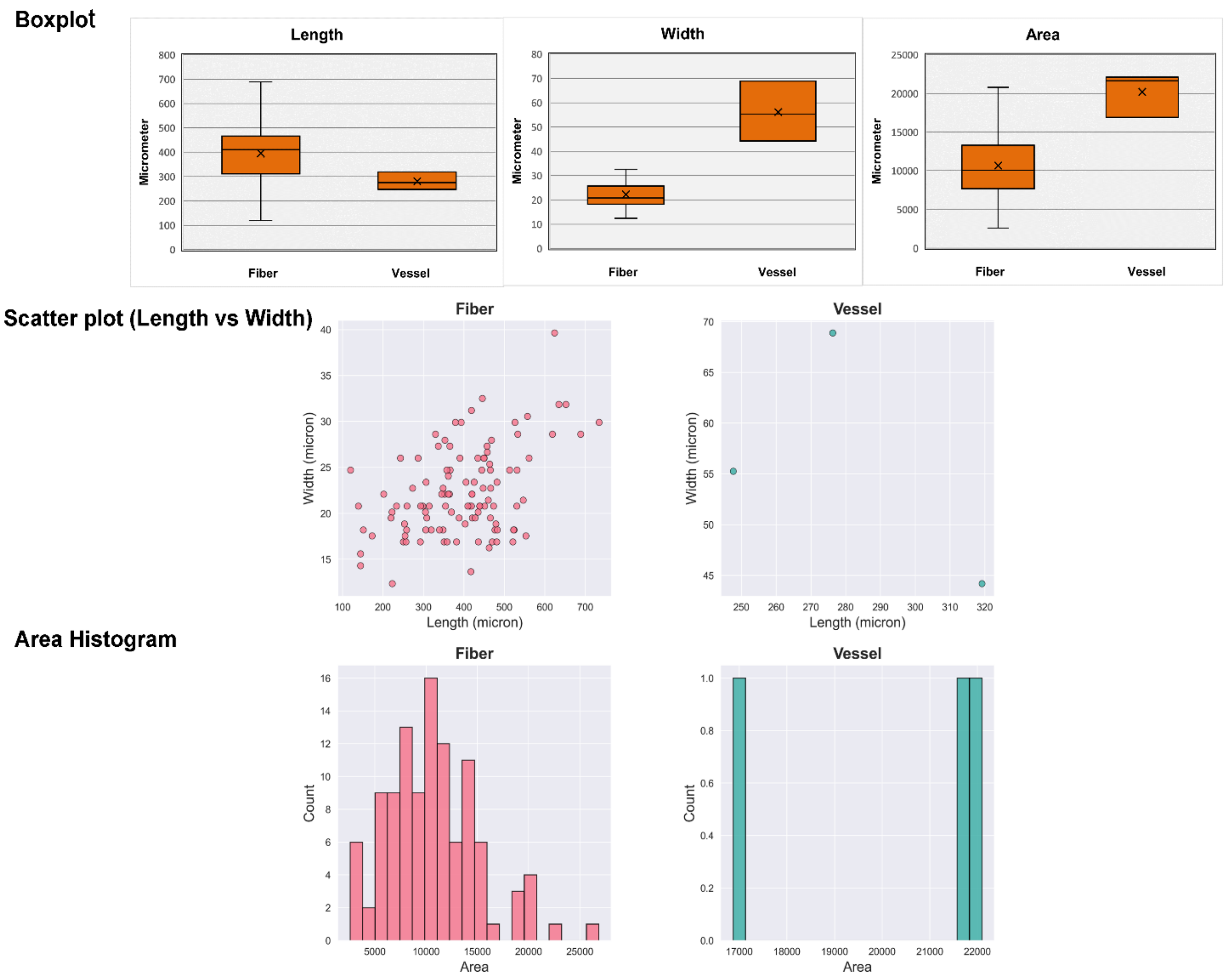

Figure S5. Analyzing fiber and vessel distributions in 2 mid-sized images 8275 x 725. The model maintains performance across different image resolutions. This scale invariance is a crucial property for real-world applications, where varying image dimensions will inevitably be encountered. The total detected fibers and vessels are 121 and 3.

How to use the model in a web application

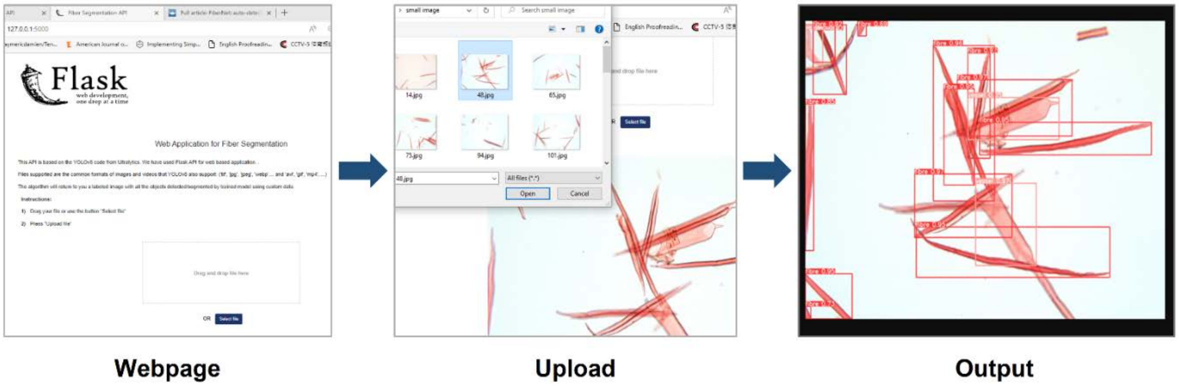

Figure S6. The image shows the steps in using the application.
